# Invasion of the Dengue Vector Aedes albopictus in the Port City of Takoradi, Southwestern Ghana

**DOI:** 10.21203/rs.3.rs-7407322/v1

**Published:** 2025-09-11

**Authors:** Faustina Adobea Owusu, Christopher Mfum Owusu-Asenso, Anisa Abdulai, Isaac Kwame Sraku, Akua Obeng Forson, Isaac Anim-Baidoo, Yaw Asare Afrane

**Affiliations:** University of Ghana; University of Ghana; University of Ghana; University of Ghana; University of Ghana School of Biomedical and Allied Health Sciences, University of Ghana; University of Ghana School of Biomedical and Allied Health Sciences, University of Ghana; University of Ghana Medical School

**Keywords:** Aedes albopictus, Aedes aegypti, Invasive species, Insecticide resistance, kdr mutations, Ghana

## Abstract

In mid-2023, *Aedes albopictus*, a key dengue vector, was unexpectedly identified during *Anopheles* surveillance in Takoradi, southwestern Ghana. *Ae. albopictus* is not known to be breeding in Ghana until this encounter. By mid-2024, the Ghana Health Service reported several outbreaks of dengue for the first time, with confirmed cases in several regions, including Takoradi. This study investigated the bionomics and insecticide susceptibility of *Ae. albopictus* through larval and adult surveys near the initial detection sites, including the seaport. Among 2,666 *Aedes* larvae collected, car tyres were the most productive habitat (66.4%). *Ae. aegypti* (87.2%) were the most abundant vector, followed by *Ae. albopictus* (12.2%). *Ae. albopictus* was fully susceptible to pyrethroids and pirimiphos-methyl, while *Ae. aegypti* was resistant to pyrethroids. PBO synergist assays restored susceptibility in *Ae. aegypti*. *kdr* mutations were detected in both species: *Ae. albopictus* had low frequencies of *F1534C* (0.18), *V410L* (0.02), *V1016I* (0.00) whilst *Ae. aegypti* showed high *F1534C* (0.72), *V1016I* (0.50), and *V410L* (0.06). These findings provide essential baseline data for public health action and necessitate the urgent need for enhanced vector surveillance and resistance monitoring in Ghana.

## Background

Over the past few decades, *Aedes albopictus*, also known as the Asian tiger mosquito, has expanded its range from its native habitats in Southeast Asia to various regions across the world including Africa^1, 2, 3^. Its invasion has been attributed to human-mediated activities such as the international trade of tyres^4^ and climate change^5, 6^, which provide ideal breeding habitats and enabling the species to establish and thrive in diverse ecological settings. The vector’s adaptability to different climatic conditions, its ecological plasticity and ability to outcompete native mosquito species has facilitated its establishment across the African continent^7^.

*Aedes albopictus* is an efficient vector of dengue fever and has been implicated in the increased outbreak of dengue and other arboviral diseases in Africa^8, 9, 10^. In West Africa, where urbanization is accelerating, the rapid invasion and establishment of the dengue vector *Ae. albopictus* poses a significant risk of outbreak and sustained transmission of arboviral diseases, particularly in densely populated cities with limited vector control programs^9^. Recent reports suggest an alarming increase in dengue cases across the sub-Saharan region; Nigeria, Côte d’Ivoire, Burkina Faso and Togo^11, 12, 13, 14^. Ghana shares its geographical borders with these countries, signifying the growing threat posed by the disease. In Ghana, after the initial discovery of a single *Aedes albopictus* at the University of Ghana Legon campus^15^, another study found *Aedes albopictus* larvae in Madina, Accra^16^. However, till date, there is paucity of data on the ecology and insecticide resistance profiles of the *Aedes albopictus* in Ghana and their potential role in arboviral disease transmission.

Interestingly, in September 2023, an entomological surveillance focused on *Anopheles* mosquitoes in Takoradi in the Western Region of Ghana led to the unexpected finding of the highly invasive vector *Aedes albopictus* in significant numbers in Apowa, a peri-urban area about 10 km away from the Takoradi port. This incidental finding may indicate the invasion and establishment of the dengue vector in the region, raising concerns about its potential role in dengue transmission. A few months later, the Ghana Health Service reported several outbreaks of dengue in Ghana for the first time in July 2024^17^.

Moreover, studies in West and Central Africa, including Côte d’Ivoire, Benin, Cameroon and the Central African Republic have reported insecticide resistance in *Ae. albopictus* populations^2, 3, 18, 19^, signaling the potential for similar challenges in Ghana.

Recognizing the increasing threat posed by *Ae. albopictus*, the World Health Organization has emphasized the need for integrated vector surveillance and control strategies to mitigate its spread^20^. In alignment with these recommendations, this study provides baseline data into the vector’s population dynamics and insecticide resistance in Takoradi, Ghana.

## Results

### Distribution and abundance of Aedes Larval Habitats.

During the study period, a total of 450 larval habitats with 50 positive breeding habitats for *Aedes* mosquitoes were identified across the study areas. The positive breeding habitats surveyed comprised of five (5) distinct habitat types, all of which were man-made. The most abundant habitat type was car tyres (62.0%) followed by discarded containers (16.0%), drums (8.0%), buckets (8.0%), and jerry cans (6.0%), (Table 1). Relative to seasons, results obtained showed that more larval habitats were encountered in the rainy season (n = 33, 66%) as compared to the dry season (n = 17, 34%) ( ^*2*^ = 3.31, *df* = 4, *P* = 0.5). All positive *Aedes* breeding habitats (n = 50, 100.0%) encountered during the study period were located outdoors (Table 1).

### Abundance of Aedes Larval in the Breeding Habitats

A total of 2,666 *Aede*s mosquito larvae were collected from the surveyed breeding habitats. The most productive habitat was car tyres 66.4% (1,771) (*F* = 1.06, *df* = 4, *P* = 0.50) (Table 2). A relatively high abundance of *Aedes* immatures were sampled during the rainy season, 65.8% (1,755), whereas 34.2% (911) were collected during the dry season (*t* = 0.04, *df* = 48, *P* = 0.96). The highest larval abundance was recorded in Apowa across both seasons (n = 2,184, 81.9%) (*F* = 2.36, *df* = 2, *P* = 0.50), (Table 2).

Larval samples collected from the study areas, which emerged into adult *Aedes* mosquitoes were morphologically identified as *Aedes aegypti* (97.3%) and *Aedes albopictus* (2.3%), (Table 3). *Aedes aegypti* larvae were collected from both study areas, while *Aedes albopictus* larvae were specifically obtained from car tires in the Takoradi Port and its surrounding areas (Table 3).

### Adult Aedes mosquito distribution

A total of 1,268 mosquitoes belonging to three different genera were sampled during the study; *Aedes* (n = 960, %), *Culex* (n = 288, %) and *Anopheles* (n = 3, %). Out of the total 960 adult *Aedes* mosquitoes sampled during the study, *Aedes aegypti* (n = 837; 87.2%) was the most predominant species by morphological identification, this was followed by *Aedes albopictus* (n = 117; 12.2%) and then *Aedes chemulpoensis* (n = 6; 0.6%). Site-specific species distribution showed that, a high abundance of *Aedes* mosquitoes were sampled in Apowa (n = 443; 46.1%) [*Aedes aegypti* (n = 371; 83.7%), *Aedes albopictus* (n = 66; 14.9%), *Aedes chemulpoensis* (n = 6; 1.4%)] (Table 4).

Significantly higher numbers of *Aedes* mosquitoes we sampled during the rainy season (n = 730; 76.0%) [Takoradi Port (n = 354; 48.5%), Apowa (n = 262; 35.9%), Anaji (n = 114; 15.6%)] (Fig. 2a) compared to the dry season n = 230; 24.0%) [Apowa (n = 181; 78.7%), Takoradi Port (n = 49; 21.3%), Anaji (n = 0; 0%)], (*t* = −2.29, *df* = 275, *P* = 0.011). Significantly high abundance of *Aedes* mosquitoes were collected outdoors (n = 909; 94.7%) as compared to indoor collection 51 (5.3%), (*t* = −5.31, *df* = 275, *P* < 0,001). Apowa recorded the highest indoor collections (n = 47; 92.6%), while most mosquitoes collected outdoor were from Takoradi Port (n = 399; 41.6%) (Fig. 2b).

A high abundance (n = 923) of host-seeking *Aedes* mosquitoes (mosquitoes sampled using HLC + BG traps) were sampled over the entire sampling period, with a high abundance (n = 858) of actively biting (mosquitoes collected using HLC) *Aedes* mosquitoes (*F* = 26.55, *P* < 0.001). (Fig. 3). Overall, a total of 37 resting mosquitoes were collected using PPK aspirators (Fig. 3).

## Insecticide Susceptibility and Synergist Assays

Phenotypic resistance results showed that the *Aedes albopictus* mosquito population was susceptible to Deltamethrin (Mortality rate (MR) = 98.8%), Permethrin (MR = 100.0%), and Pirimiphos-methyl (100.0%). In comparison, the *Aedes aegypti* population showed resistance to pyrethroids (Permethrin (74.3%); Deltamethrin (MR = 75.0%)) and full susceptibility to Pirimiphos-methyl (MR = 100.0%) ( ^*2*^ = 230, *df* = 2, *P* = < 0.001) (Fig. 4a). However, preexposure of *Aedes aegypti* mosquito to PBO before the bioassay, restored full susceptibility of the mosquito population to pyrethroids ( ^*2*^ = 79, *df* = 1, *P* = < 0.001) (Fig. 4b).

### Genotypic Resistance of Aedes mosquitoes

A sub-sample of 103 field-caught *Aedes* mosquitoes [*Ae. albopictus* = 53; *Ae. aegypti* = 50] were randomly selected and subjected to allele-specific PCR to detect the presence of *kdr* mutations *F1534C*, *V1016I* and *V410L*. Low allelic frequencies of *F1534C* (F = 0.18) ( ^2^ = 16.09, *P* < 0.001), *V410L* (F = 0.02) ( ^2^ = 0.02, *P* = 0.89) and *V1016I* (F = 0.00) were detected in *Ae*. *albopictus*. However, high allelic frequency (F = 0.66) of *F1534C* ( ^2^ = 1.26, *P* = 0.26) and *V1016I* (F = 0.50) ( ^2^ = 50, *P* < 0.001) and a much lower allelic frequency (F = 0.06) of *V410L* ( ^2^ = 0.20, *P* = 0.65) mutations was observed in *Ae. aegypti* (Table 5).

## (CC, II, LL-Kdr; FF, VV-wild-type; FC, VI, VL-Heterozygote, F; Allelic frequency, N; number)

Genotypic analysis of *Aedes albopictus* and *Aedes aegypti* mosquitoes from bioassays revealed relatively high frequencies of *F1534C* (Allelic frequency (F) = 0.75) and *V1016I* (F = 0.50) in deltamethrin-susceptible *Ae. albopictus* mosquitoes, while all three *kdr* mutations (*F1534C*, *V1016I*, and *V410L*) were present at relatively high frequencies in deltamethrin-resistant *Ae. albopictus* mosquitoes (Table 6).

Similarly, a high frequency of *F1534C* (F = 0.70) was detected in deltamethrin-susceptible *Ae. aegypti* mosquitoes, with moderate to low frequencies of *V1016I* (F = 0.40) and *V410L* (F = 0.15) respectively. Deltamethrin-resistant *Ae. aegypti* haboured relatively high frequencies of *F1534C* (F = 0.68) and *V1016I* (F = 0.50), with low frequency of *V410L* (F = 0.25). For permethrin-susceptible *Ae. albopictus*, relatively high to low levels of *V1016I* (F = 0.50) and *F1534C* (F = 0.25) was observed respectively. No *V410L* mutation was detected. In contrast, *Ae. aegypti* mosquitoes exhibited higher frequencies of all three *kdr* mutations in permethrin-susceptible and resistant populations (Table 6).

## Discussion

Due to its highly invasive nature and capability of transmitting several globally important arboviruses such as Dengue, Chikungunya, Yellow fever and Zika, the invasion of *Aedes albopictus* in Ghana poses a significant public health threat. This study showed the detection of a significant number of *Ae. albopictus* in Takoradi, and its environs implying an invasion event that may be ongoing. The invasive vector however, showed susceptibility to insecticides that could be used for its control. This study provides baseline data crucial for public health action on invasive *Ae. Albopictus* that could be crucially involved in the transmission of arboviral disease in Ghana.

The detection of the highly invasive species, *Aedes albopictus* in significant numbers in Takoradi, in the port, its environs and outside of the city represents a critical entomological finding with substantial implications for arboviral disease transmission in Ghana. Ports are well-documented entry points for invasive mosquito species^21^. The global movement of goods, via maritime transport, has been linked to the transcontinental spread of *Ae. albopictus* through used car tyres^22^. It was shown in the current study that this invasive vector is breeding more in car tires. This invasive species could spread to other parts of Ghana through transport routes that are linked to the port. Given the species’ known vector competence for dengue, chikungunya, yellow fever, and Zika viruses^23^, its establishment in Ghana poses a significant public health concern.

The emergence of *Aedes albopictus* across multiple study sites in Ghana coincided with reports of confirmed dengue outbreaks in several places in the country, including Southwestern Ghana by the Ghana Health Service^17^. While our current data do not establish a transmission link, the temporal overlap between the emergence of this invasive species and the surge in dengue cases raises critical questions. There is a big gap to ascertain potential involvement of *Ae. albopictus* in ongoing dengue transmission outbreaks. Understanding the role and extent to which this species contributes to dengue transmission is essential.

While *Ae. aegypti* remains the dominant urban vector, the introduction of *Ae. albopictus*, a competent dengue vector, could alter transmission dynamics. Its behavioral plasticity, ecological shift of the previously sylvatic vector into urban areas, and ability to exploit diverse breeding habitats enhance its adaptability and survival in diverse environments, potentially increasing its role in arboviral transmission dynamics. These traits not only allow *Ae. albopictus* to thrive under varying ecological pressures but also position it to potentially displace native *Ae. aegypti* populations. A similar trend has been reported in the Republic of Congo, where *Ae. albopictus* has overtaken *Ae. aegypti* as the dominant urban vector^24, 25,26, 27^. Such displacement, coupled with the co-circulation of multiple *Aedes* species, may intensify arboviral transmission risks and complicate control strategies in endemic settings

The WHO bioassays revealed full susceptibility of *Ae. albopictus* to pyrethroids and organophosphates. The susceptibility of these vectors may imply that insecticide-based control strategies could still yield high operational efficacy against this emerging vector, particularly in areas where it is newly established. This observation may suggest a critical advantage for effective vector control before the development and spread of resistance and establishment and expansion of *Ae. albopictus* populations. This finding was consistent with another study from Benin^18^. The low phenotypic resistance observed may be attributable to the species’ relatively minimal prior exposure to insecticides and recent urban establishment. Contrary to the finding reported for *Ae. albopictus*, *Ae. aegypti* populations were resistant to pyrethroids, with full susceptibility restored after pre-exposure to PBO, suggesting metabolic resistance mechanisms may be involved. This is consistent with prior reports in Ghana, where pre-expose of pyrethroid resistant *Aedes aegypti* to PBO, restored susceptibility^28^.

## Conclusion

This study is the first to provides a baseline assessment of the invasion of *Ae. albopictus* and its insecticide resistance status in Ghana, and highlights the significant risk posed by this vector in arboviral disease transmission. Enhanced entomological and molecular surveillance is needed at major ports of entry and high-risk urban centers in Ghana to ascertain their involvement in the ongoing dengue transmission in Ghana, as well as the extent of the invasion.

## Materials and Methods

### Study sites

**Figure 1:**
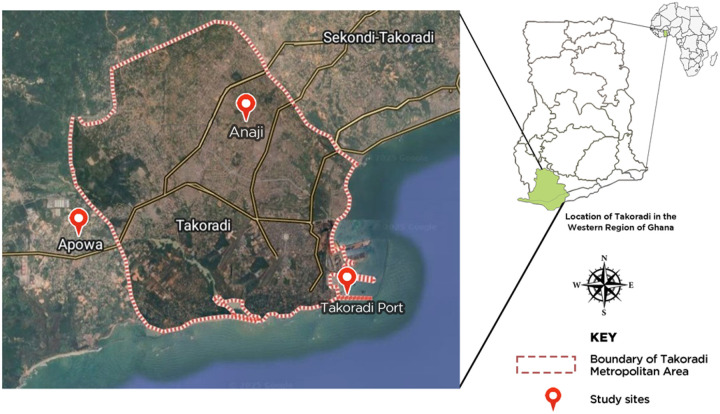
Map of Western Region of Ghana, showing the study sites

**Figure 2:**
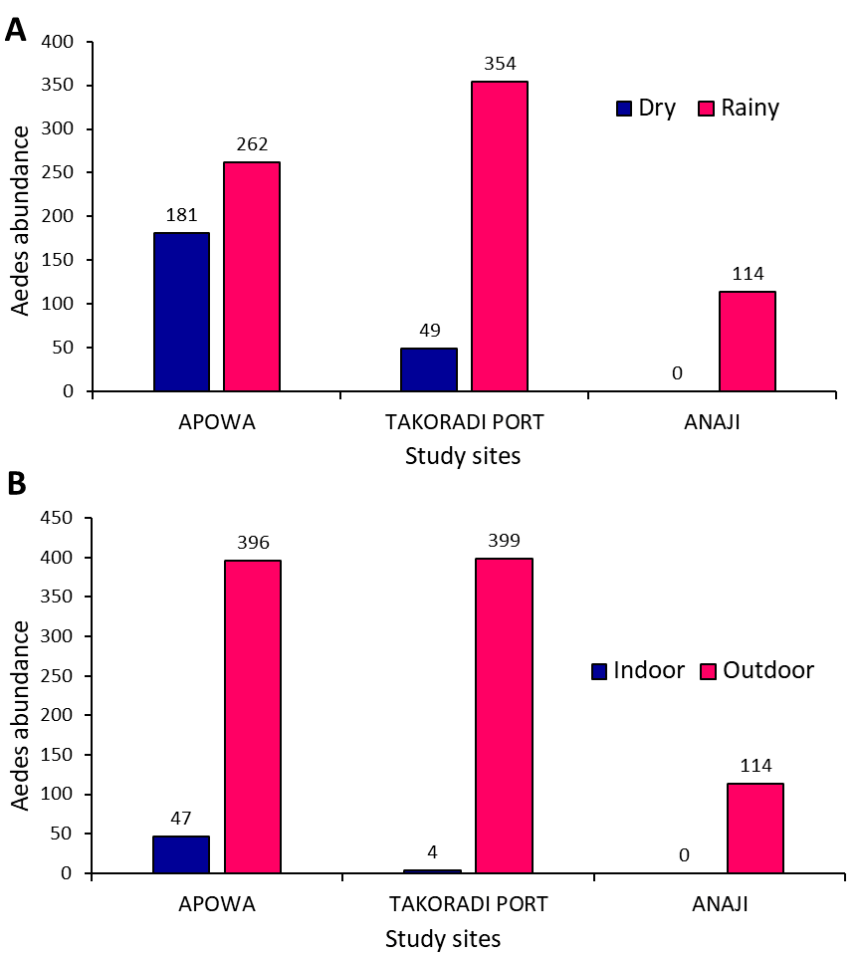
*Aedes* abundance per study site; a: seasonal abundance of Adult *Aedes* Mosquitoes; b: resting location (indoor/outdoor)

**Figure 3:**
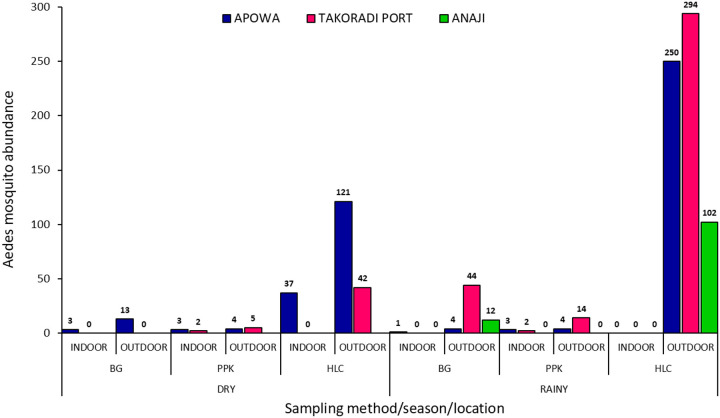
Seasonal Aedes Abundance per Trap type (BG-Biogents sentinel trap; HLC-Human Landing Catches; PPK-Prokopack aspiration)

**Figure 4:**
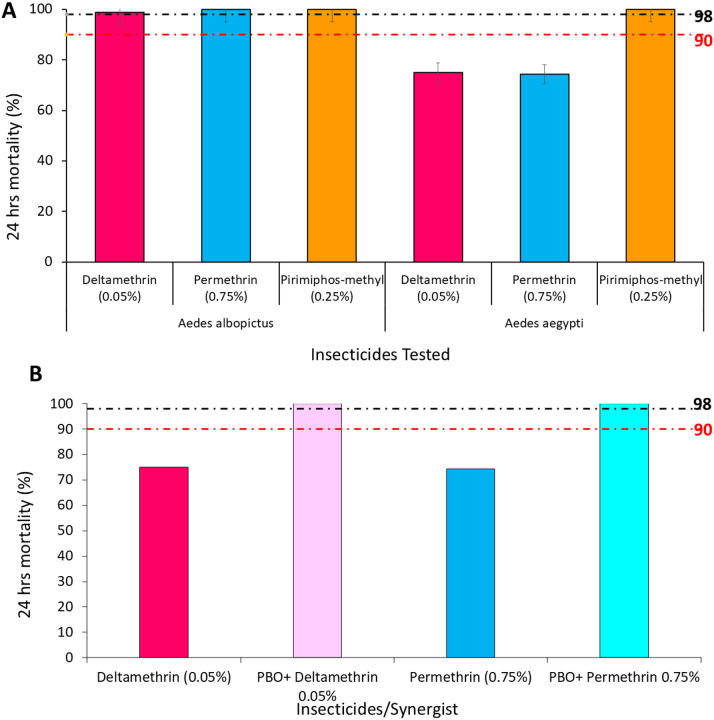
Phenotypic Resistance profile of *Aedes*to Insecticides; a: WHO susceptibility bioassay for *Ae. aegypti* and *Ae. albopictus*; b: PBO synergist assay on *Ae. aegypti*.

This study was conducted in three (3) localities Southwestern part of Ghana; Apowa (4°53’0” N, 1°49’0” W), Anaji (4°54’16” N, 2°6’51” W), and the Takoradi Port and surrounding areas (4°54’00” N, 1°44’00” W) to investigate the distribution, behavior, and insecticide resistance profiles of *Aedes albopictus*. Apowa where *Ae. albopictus* was first found accidentally in significant numbers became a focal site, while Anaji and the Takoradi port and its surrounding areas were selected to assess potential spread and introduction points of this invasive species (Fig. 1). The sea port at Takoradi receives all sorts of shipment including cars, car tyres, car spare parts, from all over the world. This may pose a substantial risk of introducing and establishing the *Ae. albopictus* and other invasive species into the port and its surrounding areas, which may increase the potential for arboviral transmission. The extensive transportation networks linking Takoradi to other regions of Ghana could facilitate the rapid spread of *Ae. albopictus* and its associated pathogens.

The accidental detection of *Ae. albopictus* was in Apowa, and was selected as a study site following the citing of a significant abundance of *Ae. albopictus* within this area. Apowa, a peri-urban area in the Ahanta West Municipal District is situated about 10 km away from the Takoradi port and 6 km from Anaji.

Anaji is a residential suburb situated within the Sekondi-Takoradi metropolitan area. Anaji lies approximately 4 km west of central Takoradi, the regional capital. Anaji is about 8 km north-west to the Takoradi port. The site was selected to ascertain the spread of the dengue vector away from the port and Apowa. Vector sampling in Anaji was done only in the rainy season due to logistics reason. From each of these sites with the exception of Anaji, both larval and adult collections were conducted during the rainy and dry seasons from September 2023 to February 2024. However, only the surrounding areas of the Takoradi port was sampled during the dry season, since the team was not able to acquire clearance to enter the port premises. After receiving clearance and collaborations with the Takoradi Port Health, the area within and around the port were sampled during the rainy season.

With an average annual temperature of 26.5°C and a mean yearly rainfall of 787 mm, the selected study areas which are located in the coastal savannah region of Southwestern Ghana, has a tropical savannah weather pattern. The region has a bimodal rainfall pattern, with the long rainy season occurring from April to June and the minor one from October to November.

This study was approved by the Ethics and Protocol Review Committee of the College of Health Sciences, University of Ghana (protocol identification number: CHS-Et/M.9-P4.3/2023–2024). All methods were carried out in accordance with relevant guidelines and regulations, including the ethical principles outlined in the Declaration of Helsinki for medical research. Meetings were held at each study site with chiefs, community leaders, and residents to introduce the research. Permission to conduct the study at the various sites was obtained from community leaders. Verbal informed consent was obtained from community leaders and residents for mosquito sampling activities.

#### Mosquito sampling and characterization of Aedes breeding habitats

Extensive larval surveys were conducted from September 2023 to February 2024 in these study sites to locate water-holding containers (e.g., tyres, jerry cans, drums) in and around human habitations and inspected for *Aedes* immatures. The habitat type, its location within a household (whether indoor or outdoor) was recorded. All potential *Aedes* breeding containers were examined for the presence of *Aedes* immature and recorded in each site. Using pipettes and ladles, immature stages (field generation, Fo) were collected from containers positive for *Aedes* mosquitoes including vehicle tyres, drums, jerry cans, tanks, buckets and abandoned containers. For each sampling site, *Aedes* immatures were pooled in plastic larval bowls and transferred to the insectary at the Department of Medical Microbiology, University of Ghana. Larvae obtained were fed with TetraMin Baby fish food (Tetra Werke, Melle, Germany) throughout their development.

Upon emergence into adults, they were morphologically identified using standard taxonomic keys^29^. Once identified as *Ae. aegypti* or *Ae. albopictus*, mosquitoes of the same species and from the same locality were pooled into separate individual cages. Due to their low abundance, *Ae. albopictus* mosquitoes were reared to their first filial generation (F_1_) for adult bioassays. At the insectary, mosquito populations were maintained under controlled environmental conditions (relative humidity: 80 ± 10%, temperature: 27 ± 2°C) and females were fed on rabbits to complete their gonotrophic cycle. The geographic coordinates of all sampling sites were documented with a geographical position system (GPSMAP^®^ 60CSx).

#### Adult Aedes mosquito collections

To determine the spatial distribution and vector behaviour of adult *Aedes* mosquitoes, sampling was conducted indoors and outdoors of houses at each site using three sampling techniques; the BG-Sentinel 2 traps (BG trap) (Biogents AG, Weissenburgstr 22, 93055 Regensburg, Germany), Human landing catches (HLC) and Prokopack Aspirators (PPK) (John W. Hock Company, Gainesville, U.S.A.). At each study site, from the hours of 4:00 pm to 7:00 pm, the BG traps were positioned indoors (in bedrooms and living rooms) and outdoors (on open verandas, or under sheds/ trees where people gather, approximately 5 meters from the home). The traps were baited with CO_2_, which was a mixture made by adding 17.5 g of yeast (Angel Yeast (Egypt) Co. Ltd.) and 250 g of sugar to 1 liter of water^30^.

For the HLC sampling technique, trained volunteers acted as both baits and catchers. Four trained volunteers (two stationed indoors and two others outdoors) collected host-seeking *Aedes* mosquitoes daily between the hours 4:00pm to 7:00pm. Prokopack aspiration was employed to mechanically aspirate indoor and outdoor resting *Aedes* mosquitoes. The *Aedes* mosquitoes collected were stored in clearly labelled paper cups after which they were transported to the insectary for identification and molecular analysis. Collected *Aedes* mosquitoes were knocked down with chloroform and preserved in well-labelled Eppendorf tubes containing silica gel. On each sampling day, previously sampled homes were not visited again to sample mosquitoes. For each sampling technique, houses were randomly selected at each site and GPS coordinates were recorded for all collection points.

#### Morphological identification of Aedes mosquito species

The different *Aedes* mosquito species that were sampled were identified morphologically using the identification keys of Huang *et al*^29^. The mosquito samples were further categorized based on sex. *Aedes albopictus* and *Ae. aegypti* were differentiated from other found *Aedes* mosquitoes by the following morphological characteristics; *Ae. albopictus*: its distinctive black and white striped pattern on the body and legs. The thorax (mid-section) has a prominent silver-white line down the middle; *Ae. aegypti*: distinctive black and white markings on their bodies, especially the lyre-shaped pattern on their thorax and white bands on their legs.

### Insecticide Susceptibility and Synergist Assays

WHO tube bioassays were conducted to assess the phenotypic resistance of F_1_ *Aedes albopictus* and Fo *Aedes aegypti* to 0.05% deltamethrin, 0.75% permethrin, and 0.25% pirimiphos-methyl, following WHO guidelines^31^. Mosquitoes (3–5 days old, non-blood-fed) were exposed for 60 minutes, with knockdown recorded every 10 minutes and mortality assessed after 24 hours. While the impregnated test papers used were designed for *Anopheles* mosquitoes, they remain widely used for *Aedes* susceptibility testing^32^.

To evaluate the role of cytochrome P450 monooxygenases in resistance, PBO synergist assays were performed. Mosquitoes were pre-exposed to 4% PBO papers for 1 hour before being transferred to permethrin (0.75%) or deltamethrin (0.05%) for another hour. Knockdown was recorded during exposure, and mortality was assessed at 24 hours. These assays were conducted following WHO standards^31^. Resistant (alive) and susceptible (dead) *Aedes* mosquitoes were stored in silica gel for further morphological identification and molecular analysis.

#### Genotyping of kdr mutations in Aedes albopictus and Aedes aegypti populations.

A sub-sample of 103 adult *Aedes* mosquitoes and 181 phenotyped pyrethroid-resistant and susceptible *Aedes* mosquitoes were genotyped for *kdr* mutations, *F1534C*, *V1016I* and *V410L*. Total DNA was extracted from whole mosquitoes using the DNeasy Tissue Kit (Qiagen, In USA). Genotyping of the *kdr* mutations was done using allele-specific PCR according to the protocols of Linss *et al*.^33^ and Villanueva-Segura *et al*.^34^.

## Data Management and Analysis

Descriptive analysis was done to visualize WHO susceptibility data, resistant allele frequencies, and mosquito species composition from the selected sites using graphs and tables.

WHO insecticide susceptibility levels were classified using the WHO criteria^35, 36^. Allele frequencies of resistance gene markers in the vector populations at each site were calculated using Hardy-Weinberg equilibrium (HWE), with the formula F (allele frequency) = (2nRR + nRS) / 2N.

Inferential statistics were applied to compare distributions across sites, seasons, and collection methods. Student’s t-tests were used to compare mean mosquito abundances between groups (e.g., dry vs. rainy season; indoor vs. outdoor), while Chi-square ( ^2^) tests were applied to assess differences in species composition and insecticide susceptibility outcomes. Statistical significance was set at *P* ≤ 0.05. All statistical analyses were done in R 4.2.2 via RStudio (2022.12.0 + 353) and STATA/IC 14.1.

## Figures and Tables

**Table 1:** Distribution and Abundance of Larval Habitats.

Habitat type	Count of habitat type		
	Dry (%)	Rainy (%)	Total (%)
Bucket	2 (50.0)	2 (50.0)	4 (100.0)
Discarded container	3 (37.5)	5 (62.5)	8 (100.0)
Drum	2 (50.0)	2 (50.0)	4 (100.0)
Jerry can	2 (66.7)	1 (33.3)	3 (100.0)
Car tyre	8 (25.8)	23 (74.2)	31 (100.0)
**Total**	**17 (34.0)**	**33 (66.0)**	**50 (100.0)**

**Table 2:** The seasonal distribution of *Aedes* larvae across the study sites.

Container type	Apowa		Takoradi Port	
	Dry (%)	Rainy (%)	Dry (%)	Rainy (%)
Drum	97 (10.6)	173 (13.6)	0 (0.0)	0 (0.0)
Discarded container	145 (15.9)	144 (11.3)	0 (0.0)	103 21.4)
Jerry can	70 (7.7)	72 (5.7)	0 (0.0)	0 (0.0)
Car tyre	583 (64.0)	809 (63.6)	0 (0.0)	379 (78.6)
Bucket	16 (1.8)	75 (5.9)	0 (0.0)	0 (0.0)
**Total**	**911 (100.0)**	**1,273 (100.0)**	**0 (0.0)**	**482 (100.0)**

**Table 3:** Morphological Identification of Emerged *Aedes* Mosquitoes

Study sites	*Ae. aegypti* (%)	*Ae. albopictus* (%)	Total (%)
Apowa	460 (100.0)	0 (0.0)	460 (100.0)
Takoradi Port	250 (92.6)	20 (7.4)	270 (100.0)
**Total**	**710 (97.3)**	**20 (2.7)**	**730 (100.0)**

**Table 4:** Morphological Identification of Adult *Aedes* Abundance

Study sites	*Ae. aegypti* (%)	*Ae. albopictus* (%)	*Ae. chemulpoensis* (%)	Total (%)
Apowa	371 (83.7)	66 (14.9)	6 (1.4)	443 (100.0)
Takoradi Port	352 (87.3)	51 (12.7)	0 (0.00)	403 (100.0)
Anaji	114 (100.0)	0 (0.00)	0 (0.00)	114 (100)
**Total**	**837 (87.2)**	**117 (12.2)**	**6 (0.6)**	**960 (100)**

**Table 5:** Number of genotypes and frequencies of the *V1016I*, *F1534C* and *V410L* mutations in the voltage-gated sodium channel gene of adult *Aedes aegypti* and *Aedes albopictus* mosquitoes.

	N	*F1534C*				*V410L*				*V1016I*			
Species		CC	FF	FC	F	LL	VV	VL	F	II	VV	VI	F
*Aedes albopictus*	53	6	40	7	0.18	0	51	2	0.02	0	53	0	0.00
*Aedes aegypti*	50	20	4	26	0.66	0	44	6	0.06	0	0	50	0.50
Total	103	26	44	33		0	95	8		0	53	50	

**Table 6:** Allelic frequencies of the V1016I, F1534C and V410L mutations in the voltage-gated sodium channel gene of *Aedes aegypti* and *Aedes albopictus* mosquitoes from WHO Bioassays.

Insecticide	Species			Resistant genes (F)		
		Phenotype	N	F1534C	V410L	V1016I
**Deltamethrin**	*Ae. albopictus*	S	30	0.75	0.03	0.50
		R	1	0.50	0.50	0.50
	*Ae. aegypti*	S	30	0.70	0.15	0.40
		R	30	0.68	0.25	0.50
**Permethrin**	*Ae. albopictus*	S	30	0.25	0.00	0.50
		R	0	0	0	0
	*Ae. aegypti*	S	30	0.82	0.73	0.50
		R	30	0.87	0.60	0.50

(S; Susceptible, R; Resistant, N; number, F; Allelic frequency)

## Data Availability

The datasets generated during and/or analysed during the current study are available from the corresponding author on reasonable request.
